# Benefits of osimertinib treat a lung adenocarcinoma patient with germline *EGFR* T790M, somatic *EGFR* 19-Del, *TP53* and *PIK3CA* mutations

**DOI:** 10.1186/s13053-024-00286-4

**Published:** 2024-08-19

**Authors:** Yingxue Li, Guangqi Li, Zheng Zheng, Wenjuan Wen, Haihui Zhao, Xia Liu, Jiaping Xie, Lin Han

**Affiliations:** 1 Department of Pathology, Liaocheng People’s Hospital, Liaocheng, 252000 Shandong People’s Republic of China; 2 Department of Pathology, School of Basic Medicine Science, Shandong University, Jinan, 250012 Shandong People’s Republic of China; 3 Department of Pathology, The Affiliated Hospital of Qingdao University, Qingdao, 266000 Shandong People’s Republic of China; 4 Department of Gastroenterology, Liaocheng People’s Hospital, Liaocheng, 252000 Shandong People’s Republic of China; 5 Department of Gastroenterology, The Fifth People’s Hospital of Liaocheng, Liaocheng, 252000 Shandong People’s Republic of China

**Keywords:** Germline T790M mutation, Somatic *EGFR* 19-Del mutations, *PIK3CA* mutation, Lung adenocarcinoma, Osimertinib

## Abstract

**Background:**

Somatic mutations in the *EGFR* gene occur in about 50% of non-small cell lung cancers, with the T790M mutation significantly contributing to secondary resistance against *EGFR*-TKI drugs. However, *EGFR* T790M germline mutations rarely occur.

**Case presentation:**

In this study, we report a case of a lung adenocarcinoma family lineage linked to a germline *EGFR* T790M mutation. The main subject was diagnosed with stage IV lung adenocarcinoma and experienced a 19-month period without disease progression while treated with Osimertinib. We collected both clinicopathological and familial data from a patient with lung adenocarcinoma. Next-generation sequencing of 40 key genes was performed on the proband’s tumor tissue. To detect *EGFR* germline mutations, Sanger sequencing was conducted on peripheral blood mononuclear cells from the proband and his two daughters. Mutations such as *EGFR* T790M, *EGFR* 19-Del, *TP53*, and *PIK3CA* were identified in the proband’s lung cancer tissue. Additionally, germline *EGFR* T790M mutations were confirmed in the proband and his daughters through sequencing of their peripheral blood samples. CT scans revealed multiple pulmonary nodules in both daughters.

**Conclusions:**

These observations suggest that germline mutations in *EGFR* T790M could be strongly linked to a familial predisposition to lung cancer.

**Supplementary Information:**

The online version contains supplementary material available at 10.1186/s13053-024-00286-4.

## Background

The somatic mutation rate of the *epidermal growth factor receptor* (*EGFR*) gene is approximately 50% in Asian populations with lung adenocarcinoma and around 30% in Western populations. The most prevalent *EGFR* somatic mutations are the single point mutation in exon 21 (L858R) and the deletion in exon 19 (Del-19), which together constitute about 90% of all *EGFR* mutations [1]. These mutations are linked to increased sensitivity to *EGFR* tyrosine kinase inhibitors (TKIs), making *EGFR* TKIs a primary treatment option for non-small cell lung cancer (NSCLC) patients [2–4]. However, almost all patients eventually develop resistance to *EGFR*-TKIs within a year of treatment, predominantly due to secondary *EGFR* T790M point mutations. Primary germline *EGFR* T790M mutations, on the other hand, are rarely reported [5, 6].

*EGFR* germline mutations have been detected in families with a history of lung adenocarcinoma, offering potential insights into the risk and carcinogenic mechanisms of lung cancer. Despite this, due to the absence of robust screening methods, only a limited number of affected families have been identified since the discovery of the *EGFR* germline mutation [7–15].

In a study conducted in Asia, only five out of 427 lung adenocarcinoma cases exhibited a primary de novo T790M mutation, with one confirmed as a germline T790M mutation through sequencing of a peripheral blood sample [16]. Similarly, an American study involving 369 lung adenocarcinoma patients who had never smoked found two cases of germline T790M mutations. These included one Asian-origin female and one Eastern European male [17].

Treatment for newly diagnosed advanced NSCLC patients that is based on their molecular status is regarded as the standard approach. However, there is no consensus on the treatment for familial hereditary lung cancer [2]. Multiple *EGFR* germline mutations have been identified in lung cancer patients [7–15], but the optimal treatment regimen for lung cancer with *EGFR* germline mutations is unknown.

In this brief report, we describe the case of a Chinese male diagnosed with lung adenocarcinoma, harboring both a germline *EGFR* T790M mutation and concurrent somatic mutations in *EGFR* 19-Del, *TP53*, and *PIK3CA*. Following successful treatment with Osimertinib, he achieved a progression-free survival (PFS) of 19 months.

## Case presentation

### Clinical pathology and pedigree data collection

**Fig. 1:**
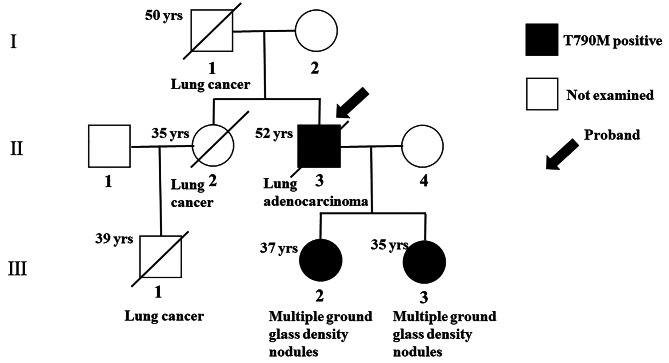
Pedigree of family of the proband (II-3). Boxes and circles indicate males and females, respectively; numbers at left above indicate age at death or time of mutation analysis; and oblique line shows deceased family members

**Fig. 2:**
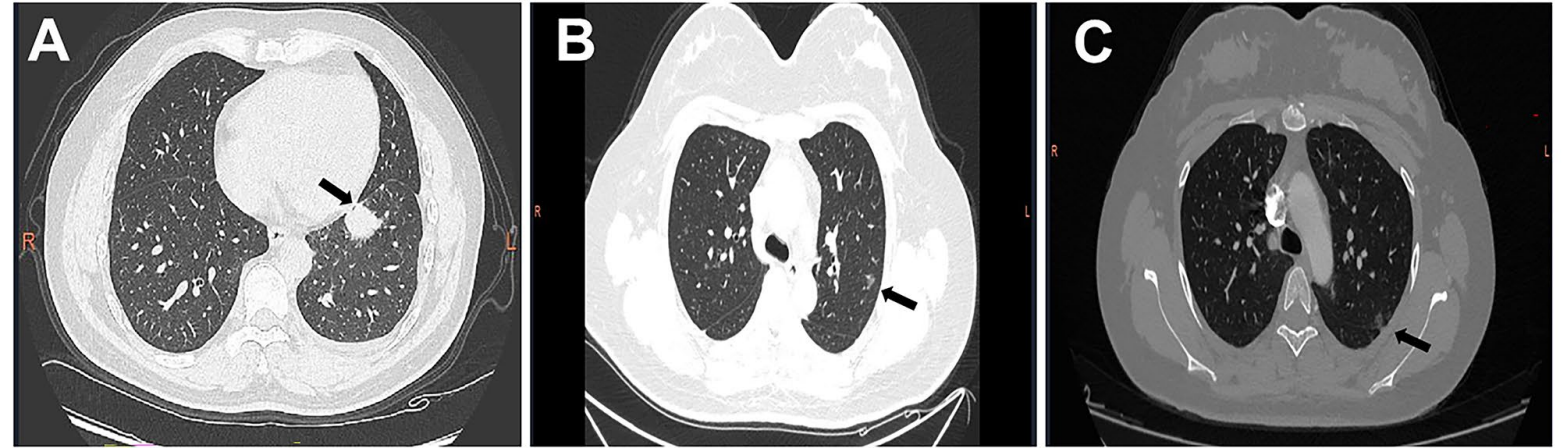
CT scans of the proband and his two daughters. **A** Adenocarcinoma(arrow) of the left upper lobe of the proband(II-3). **B** Double-lung ground-glass nodules (arrows) of the proband’s eldest daughter(III-2). **C** Double-lung ground glass nodules (arrows) of the proband’s youngest daughter(III-3)

The case involved a 52-year-old man (II-3, Fig. 1) who initially presented with hemoptysis. A chest CT scan showed multiple pulmonary nodules in his left lung (Fig. 2A). Diagnostic bronchoscopy of the right lung’s lower lobe confirmed lung adenocarcinoma. Thoracoscopic surgery included resection of a tumor in the left lower lobe and biopsies of chest wall nodules. Multiple nodules were found in the chest wall, visceral pleura, and diaphragm during surgery, along with a small volume of yellowish hydrothorax. The final pathological assessment determined the cancer to be stage IVA (T1cNxM1b) lung adenocarcinoma [18]. One month post-surgery, the patient underwent five cycles of chemotherapy with Pemetrexed disodium and Lobaplatin. Despite initial treatment, tumor progression occurred one year after surgery. Subsequently, Osimertinib was administered for targeted therapy, yet the patient developed brain metastases after 19 months. After four cycles of anti-tumor treatment with ameritinib combined with bevacizumab, the patient’s condition progressed, leading to his death. The patient’s overall survival from the diagnosis of stage IV lung cancer was 42 months.

His father and a sister (I-1 and II-2, respectively; Fig. 1) died of lung cancer. Additionally, his nephew (III-1, Fig. 1) passed away from lung cancer at the age of 35. As they resided in different provinces, the histological types and mutation statuses of their tumors were not determined. The patient’s two daughters (III-2 and III-3, respectively; Fig. 1) were found to have multiple ground-glass density nodules in both lungs during CT scans at a physical examination (Fig. 2B-C). However, all nodules were smaller than 1 cm in diameter and did not necessitate surgical intervention.

### Tumor and germline genotyping

Formalin-fixed paraffin-embedded lung adenocarcinoma tissue from proband was selected, tumor cell content was about 70%, and DNA and RNA were extracted using FFPE DNA / RNA extraction kit (Amoy Dx, Xiamen, China). A sequencing library was then constructed using a human cancer multigene mutation detection kit from the same company, designed for high-throughput sequencing. This library allowed for the detection of mutations in 40 genes including *EGFR*, *KRAS*, *BRAF*, *ERBB2*, *MET*, *ALK*, *ROS1*, *RET*, *NRAS*, *PIK3CA*, among others, as listed in Supplementary Material, Table 1. The sequencing was performed on an Illumina NextSeq 550 Dx sequencer. Peripheral blood was also collected from the proband and his two daughters; DNA was extracted from their peripheral blood mononuclear cells using a fresh blood DNA extraction kit (Amoy Dx). Germline EGFR mutations were analyzed using an ABI 3500 Dx sequencer, focusing on exons 18, 19, 20, and 21 of *EGFR*. Primer sequences can be found in the Supplementary Material (Table 2). Informed consent was obtained from the proband and all participating family members, with approval by the Ethics Committee of Liaocheng People’s Hospital (NO. 2024086).

### Pathological results

**Fig. 3:**
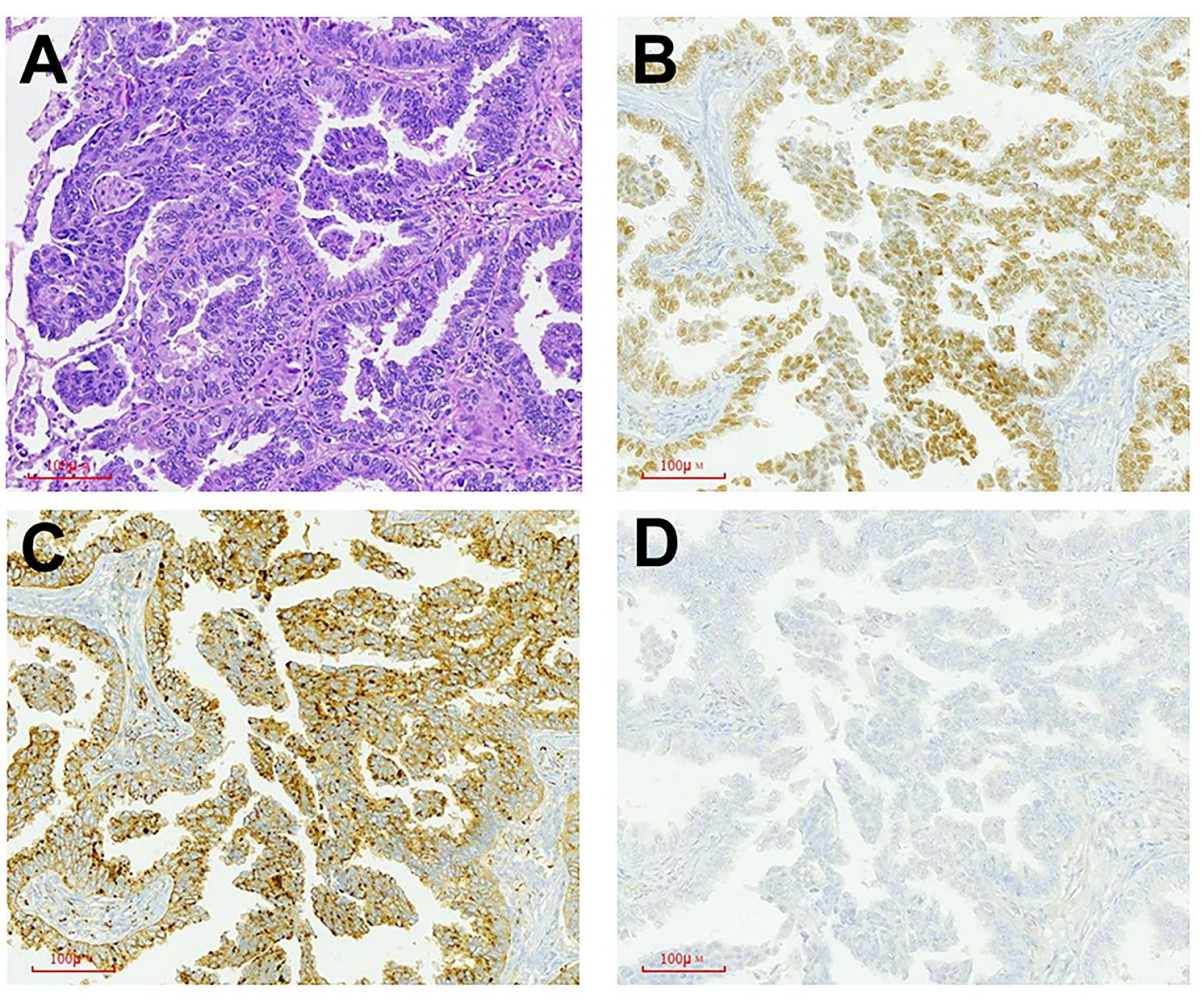
The pathological images of invasive adenocarcinoma in the proband. **A** Hematoxylin and eosin staining (200×), most of the tumor cells are adenoid and locally in solid sheets. **B** TTF-1 immunohistochemistry (200×), nuclear positivity of tumor cells. **C** NapsinA immunohistochemistry (200×) cytoplasm positivity of tumor cells. **D** P63 immunohistochemistry (200×), nuclear negative of tumor cells

Pathological examination of the proband’s excised left lung’s lower lobe revealed two nodules. The majority of the tumor cells displayed an adenoid configuration with some areas appearing in solid sheets. Immunohistochemical (IHC) staining was positive for TTF-1 and Napsin A, but negative for P63 (Fig. 3A-D). These IHC findings confirmed the diagnosis of pulmonary invasive adenocarcinoma, predominantly of the acinar type. Additionally, adenocarcinoma was observed in nodules on the chest wall. The tumor had invaded the pleural layers of the lung, and multiple intraductal tumor plugs were identified.

### Genotyping of the tumor tissues

**Fig. 4:**
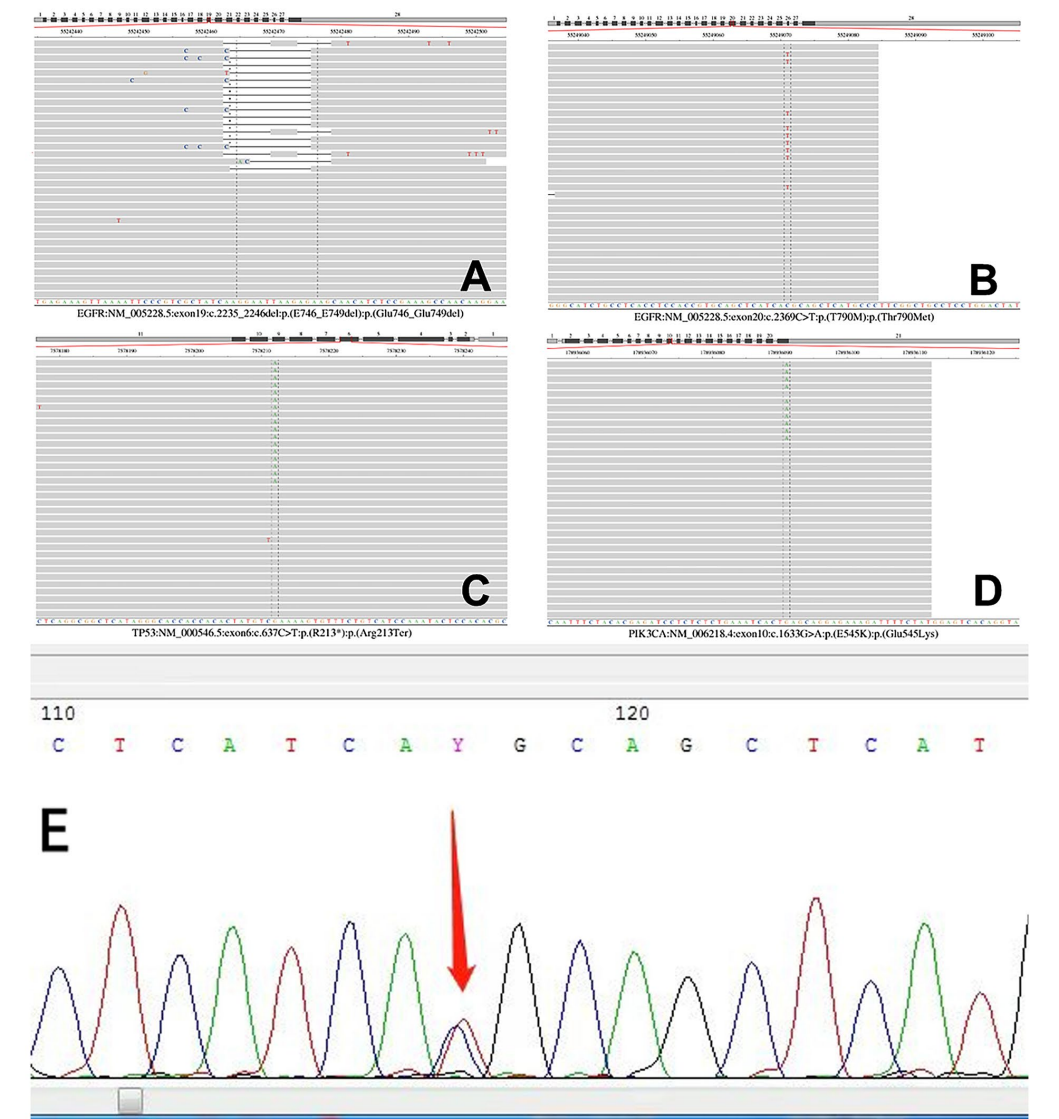
Next generation sequencing of proband lung adenocarcinoma tissue and sanger sequencing of peripheral blood mononuclear cells from the proband and his two daughters. **A-D** The *EGFR* 19Del, T790M, *TP53* and *PIK3CA* mutations were detected in the lung adenocarcinoma tissue of the proband. **E** Peripheral blood mononuclear cells from the proband and his two daughters exhibited the *EGFR* T790M mutation

*EGFR* exon20 c.2369 C > T p.(T790M), *EGFR* exon19 c.2235_2246del p.(E746_E749del), *TP53* exon6 c.637 C > T p.(R213*) and *PIK3CA* exon10 c.1633G > A p.(E545K) mutations were detected in lung adenocarcinoma tissue samples from the proband. The respective mutation frequencies were 56.54%, 48.83%, 47.80%, and 0.52% (Fig. 4A-D).

### Germline *EGFR* T790M mutations

Peripheral blood mononuclear cells from the proband and his two daughters all displayed the *EGFR* T790M mutation in exon 20 (Fig. 4E). No mutations were found in exons 18, 19, and 21 of the *EGFR* gene in any of the samples tested.

## Discussion

The *EGFR* T790M (c.2369 C > T) mutation leads to an amino acid change in exon 20 from threonine (T) to methionine (M). This mutation is found in 2% of lung cancers, with the germline form occurring in 1% of cases [19]. According to data from OrigiMed laboratory, the prevalence of EGFR T790M germline mutations in Chinese patients with NSCLC is lower than that observed in Japanese and American populations (0.08% vs. 0.54%) [17]. Preclinical studies have indicated that the T790M mutation increases *EGFR* kinase activity, thereby providing a mild proliferative impetus for lung cancer development [20]. Typically, lung cancers with T790M germline mutations are predominantly found in females, are mostly adenocarcinomas, and occasionally multifocal. Additionally, 73% of tumors with the T790M germline mutation also have other activating mutations in the *EGFR* gene. These findings are all consistent with our case report. We describe a male patient with lung adenocarcinoma who possessed the germline *EGFR* T790M mutation along with somatic mutations in *EGFR* 19-Del, *TP53*, and *PIK3CA*. This specific combination of mutations is extremely rare, with the first reported case of a patient having both germline *EGFR* T790M and somatic *EGFR* L858R and *PIK3CA* mutations documented by Lammers et al. [21].

*EGFR* T790M mutations are found in approximately 50% of patients who develop resistance to *EGFR*-TKIs, and these mutations are linked to acquired resistance to these therapies. Osimertinib, a third-generation *EGFR* TKI, effectively inhibits both *EGFR* TKI-sensitive and resistant *EGFR* T790M mutations in human NSCLC models. The US Food and Drug Administration (FDA) has approved Osimertinib for the treatment of metastatic NSCLC patients positive for the *EGFR* T790M mutation. Additionally, Osimertinib can be administered as a first-line treatment in patients with mutant *EGFR* lung cancer, either during initial *EGFR* TKI treatment or after disease progression [2, 22–24]. In our report, the proband achieved a 19-month progression-free survival using Osimertinib following postoperative progression of stage IV lung cancer. Subsequently, the patient was treated with targeted therapies including ameritinib and bevacizumab, ultimately extending survival to 42 months.

*TP53* is the most commonly co-occurring mutation in NSCLC, with approximately 60% of patients harboring *EGFR* mutations also presenting *TP53* mutations. These *TP53* mutations are often linked to resistance against *EGFR-TKIs* in Chinese patients with NSCLC. *TP53* mutations diminish the efficacy of TKIs in patients with *EGFR*-mutant NSCLC and are associated with a poorer prognosis, particularly for those with non-disruptive, non-missense mutations, and mutations outside the DNA-binding domain (DBD), specifically in exons 6 and 7. Thus, TP53 mutations serve as an independent predictor of outcomes in NSCLC patients treated with first-generation TKIs. Patients with both *EGFR* and *TP53* mutations typically have a median progression-free survival (PFS) of only 6.5 months and a median overall survival (OS) of 28.0 months, nearly half that of controls [25, 26]. In our report, co-mutations of *EGFR* 19 Del, T790M and *TP53* exon6 c.637 C > T p.(R213*) were detected in the tumor tissue of the proband. He achieved a PFS of approximately 19 months after treatment with the third-generation *EGFR*-TKI, Osimertinib, and an OS of 42 months. His prognosis exceeded those commonly reported in the literature.

*Phosphoinositide 3-kinases* (*PI3Ks*) play an important role in tumor development and progression, and recurrent activating mutations in the p110 α subunit (*PIK3CA*) have been found in some tumors. Several PI3K inhibitors have been approved for use in both solid tumors and hematological malignancies. The FDA has sanctioned the use of Alpelisib in combination with fulvestrant for treating *HR+/HER2*- advanced or metastatic breast cancer patients who have *PIK3CA* gene mutations [27]. In an elegant study reported by Lammers et al., a patient with both a germline *EGFR* T790M mutation and somatic *EGFR* L858R and *PIK3CA* mutations received a combination treatment of erlotinib 150 mg daily for four months, afatinib 40 mg daily, and cetuximab 500 mg/m2 bi-weekly for three months, along with a PI3K inhibitor for another three months. The patient maintained a good quality of life 23 months post-diagnosis of stage IV lung adenocarcinoma [21]. In our study, the *PIK3CA* exon10 c.1633G > A p.(E545K) mutation was found in the tumor tissue of the proband, but the mutation abundance was only 0.52%, so he was not treated with *PIK3CA* inhibitor. Unfortunately, Unfortunately, after disease progression following Osimertinib treatment, we were unable to obtain further tumor tissue for genetic analysis to identify changes in resistance genes. If the tumor presents with high mutation abundance *PIK3CA* mutations after Osimertinib resistance, patients will potentially benefit from *PIK3CA* inhibitor therapy [27].

When a patient exhibits more than two nodules, they are diagnosed with multiple ground-glass nodules (mGGNs), and these are classified as synchronous multiple primary lung cancer (SMPLC) when found in the lungs. With advancements in chest CT imaging technology, the incidence of mGGNs has risen sharply in recent years. mGGNs are more commonly observed in female non-smokers and may have a higher familial risk of malignancy compared to typical lung cancer patients [18, 28, 29]. In our study, the proband, who carried the *EGFR* T790M germline mutation, had multiple nodules in his left lung. Similarly, his two daughters, also carriers of the *EGFR* T790M germline mutation, showed multiple ground-glass density nodules in both lungs on their CT scans during a physical examination. Germline *EGFR* T790M mutations are exceptionally uncommon. For instance, none were found in a study involving 627 Japanese lung cancer patients [30, 31]. In a separate investigation among 31,906 Chinese lung cancer patients, only two instances of T790M germline mutations were identified. Among other *EGFR* germline mutations discovered, the G863D mutation was the most frequent, followed by P848L, D1014N, K757R, V897A, and R831H [32]. Thus, identifying these *EGFR* germline mutations is crucial for screening family members for potential cancer risks. Oxnard et al. recommended that any lung cancer patients with initial tumor tissue testing positive for *EGFR* T790M mutations should also have their peripheral blood samples retested to rule out the presence of germline mutations of *EGFR* T790M [33]. Although *EGFR* germline mutations are not typically included in routine screenings, there are several strategies to identify potential *EGFR* germline mutations during regular testing procedures. Firstly, it is crucial to thoroughly gather family history, especially of lung cancer, and perform EGFR and other germline gene tests for those patients who have a familial history. Secondly, the detection of specific mutations such as *EGFR* T790M, R776H, or V843I during pre-treatment genotyping for lung cancer may indicate a possible germline mutation. Finally, if repeated genetic tests on tumor tissue or peripheral blood ctDNA show a consistent mutation abundance of 50% for *EGFR* T790M, it is likely indicative of a germline mutation, and testing for *EGFR* germline mutations is recommended [10]. As mutation screening becomes more comprehensive and testing methods improve in sensitivity, an increasing number of patients are likely to be identified with suspected inherited or germline mutations. Crucially, these findings can impact not only the care of the patients but also their family members. Although there are no established guidelines for screening lung cancer in asymptomatic individuals with known *EGFR* germline mutations, it is recommended that family members who have been diagnosed with *EGFR* germline mutations undergo regular monitoring through CT scans.

## Conclusion

In conclusion, we have reported the case of a rare *EGFR* T790M germline mutated patient with confirmed family history developing lung cancer who achieved clinical benefit from Osimertinib treatment. This study This case underscores the importance of detecting germline mutations in the EGFR gene before treatment in lung cancer patients. It also offers important insights for treatment strategies in patients who possess both germline EGFR T790M and somatic EGFR 19-Del mutations. Future research and clinical trials are essential to develop more effective therapies for these patients.

### Electronic supplementary material

Below is the link to the electronic supplementary material.

Supplementary Material 1

## Data Availability

All data generated or analysed during this study are included in this published article.

## Abbreviations

*EGFR*: Epidermal growth factor receptor
NSCLC: Nonsmall-cell lung cancer
PFS: Progression-free survival
FDA: Food and Drug Administration
*PI3Ks*: Phosphoinositide 3-kinases
mGGNs: Multiple ground-glass nodules
SMPLC: Synchronous multiple primary lung cancer
