# Supplementary material for: Benefits of osimertinib treat a lung adenocarcinoma patient with germline EGFR T790M, somatic EGFR 19-Del, TP53 and PIK3CA mutations
Source: Hered Cancer Clin Pract. 2024 Aug 19;22:13. doi: 10.1186/s13053-024-00286-4 (PMC11331667; doi:10.1186/s13053-024-00286-4)
Supplement: Supplementary file 1 — Supplementary Material 1 [file 13053_2024_286_MOESM1_ESM.docx]

**Supplementary material:**

Table 1 List of genes from the multigene mutation detection kit for human cancer

| *ALK* | *BRAF* | *EGFR* | *ERBB2* | *KRAS* |
| --- | --- | --- | --- | --- |
| *MET* | *RET* | *ROS1* | *NRAS* | *PIK3CA* |
| *AKT1* | *CDK4* | *CTNNB1* | *DDR2* | *DPYD* |
| *ESR1* | *FGFR1* | *FGFR2* | *FGFR3* | *FGFR4* |
| *HRAS* | *IDH1* | *IDH2* | *KEAP1* | *KIT* |
| *MAP2K1* | *MYC* | *NFE2L2* | *NKX2-1* | *NRG1* |
| *NTRK1* | *NTRK2* | *NTRK3* | *PDGFRA* | *POLE* |
| *PTEN* | *RB1* | *STK11* | *TP53* | *UGT1A1* |

Table 2 Primer sequences for EGFR exon 18, exon 19, exon 20, and exon 21

| exon 18 | F | GAGGTGACCCTTGTCTCTGTGT |
| --- | --- | --- |
|  | R | CCCAAACACTCAGTGAAACAAA |
| exon 19 | F | CAATATCAGCCTTAGGTGCGG |
|  | R | GATGTGGAGATGAGCAGGGTC |
| exon 20 | F | GTCCATGTGCCCCTCCTTCT |
|  | R | TTCCCTGATTACCTTTGCGA |
| exon 21 | F | CCTCACAGCAGGGTCTTCTC |
|  | R | CCTGGTGTCAGGAAAATGCT |
